# Design of an Intelligent MEMS Safety and Arming Device with a Condition Feedback Function

**DOI:** 10.3390/mi14061130

**Published:** 2023-05-27

**Authors:** Kexin Wang, Tengjiang Hu, Yulong Zhao, Wei Ren, Yifei Wang

**Affiliations:** 1 State Key Laboratory for Manufacturing Systems Engineering, Xi’an Jiaotong University, Xi’an 710049, China; wkx741565@stu.xjtu.edu.cn (K.W.); zhaoyulong@xjtu.edu.cn (Y.Z.); wangyif@stu.xjtu.edu.cn (Y.W.); 2 Science and Technology on Applied Physical Chemistry Laboratory, Shaanxi Applied Physical Chemistry Research Institute, Xi’an 710061, China; rw0192@163.com

**Keywords:** safety and arming device, electrothermal actuator, bistable mechanism, condition feedback, MEMS

## Abstract

A safety and arming device with a condition feedback function has been designed in this article to improve the intelligence and safety of ignition devices. The device achieves active control and recoverability by virtue of four groups of bistable mechanisms which consist of two electrothermal actuators to drive a semi-circular barrier and a pawl. According to a specific operation sequence, the barrier is engaged by the pawl at the safety or the arming position. The four groups of bistable mechanisms are connected in parallel, and the device detects the contact resistance generated by the engagement of the barrier and pawl by the voltage division of an external resistor to determine the parallel number of the mechanism and give feedback on the device’s condition. The pawl as a safety lock can restrain the in-plane deformation of the barrier in the safety condition to improve the safety function of the device. An igniter (a NiCr bridge foil covered with different thicknesses of Al/CuO films) and boron/potassium nitrate (B/KNO_3_, BPN) are assembled on both sides of the S&A device to verify the safety of the barrier. The test results show that the S&A device with a safety lock can realize the safety and arming functions when the thickness of the Al/CuO film is set to 80 μm and 100 μm.

## 1. Introduction

Safety and Arming (S&A) devices are vital to ensuring weapon systems’ safety, reliability, and lethality by isolating sensitive igniters from insensitive charges through a removable barrier mechanism to control their energy transfer (allowing standard ignition and preventing accidental ignition). With the demand for miniaturization and intelligence in modern information warfare, MEMS S&A devices have become a focus of research due to their outstanding features such as micromechanics, integrated structure, and intelligent actuation [1,2]. Existing MEMS S&A devices already have miniaturized structures and different drive principles, but they are still insufficient in terms of intelligence.

The intelligence of S&A devices is reflected in their active control, recoverability, and condition feedback. Actively controlled S&A devices can be armed in arbitrary environments, whereas traditional S&A devices use ammunition-specific environmental information for one-time passive arming. Initially, Charles H. Robinson proposed an inertial-driven micro S&A device for small caliber munition, which is armed by setback load and fabricated by the UV-LIGA process [3]. Similarly, Seok et al. and Jeong et al. presented two miniature S&A devices armed with setback and centrifugal loads [4,5]. In addition, Lou et al. designed a micro S&A device for a small caliber projectile that is armed by centrifugal loads [6,7]. Recently, Lei et al. analyzed the mechanical responses of an inertially driven S&A device under dual-environment inertial loads [8]. Inertially driven S&A devices are the most reliable, but they are inflexible on a complicated battlefield. For novel unmanned combat platforms, such as unmanned aerial vehicles (UAV), the ammunition lacks an inertial environment and does not need to be activated on every mission. Therefore, armed S&A devices must be able to recover to a safe condition to ensure the safety of the platform’s recycling. To ensure the success of each drive, S&A devices urgently need a simple and reliable condition feedback function. Electrically driven S&A devices are armed by electrical signals, such as electromagnetic and electrothermal signals. For example, Hu et al. designed an electrothermal S&A device that can control the barrier’s movement with the cooperation of four electrothermal actuators [9,10]. In addition, Maurer et al. proposed an electromagnetic S&A device in which the barrier is pulled by an electromagnetic coil [11,12] and Sun et al. presented an electromagnetic coil used in MEMS S&A devices [13]. All the electrically driven S&A devices can achieve active control and recoverability through bistable mechanisms, but no articles have been published on the condition feedback function of MEMS S&A devices.

Herein, we propose the design and characterization of an intelligent MEMS S&A device with a condition feedback function, which is an improvement of an electrothermal bistable S&A device [14,15,16]. This intelligent S&A device has a smaller size (5.8 × 5.8 mm^2^), lower drive voltage (11 V), and higher safety (barrier safety lock) through an optimized structural layout. The device uses two actuators to control a pawl and a barrier so that they can engage or disengage to realize the bistability of the barrier and test the pawl and barrier contact to achieve the feedback function. The S&A device is then assembled in an ignition device to test the safety improvement of the safety lock.

## 2. Modeling

### 2.1. Structure of the Ignition Device

**Figure 1:**
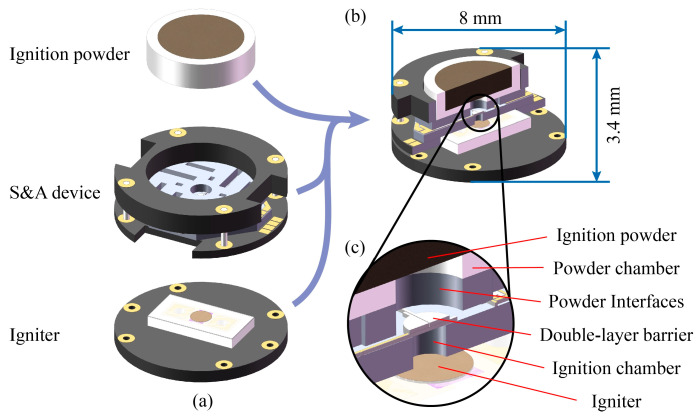
Structure of the ignition device. (**a**) Key components, (**b**) section view, and (**c**) detailed view.

The S&A device is designed as an ignition device by assembling an igniter and an ignition powder on both sides, the structure of which is improved based on the previous research, with an overall size of Φ8 × 3.4 mm^3^, as shown in Figure 1. The center of the S&A device is a double-layer barrier structure with interfaces at the top (diameter of 500 μm) for connecting the ignition powder and at the bottom (diameter of 1000 μm) for connecting the igniter, as shown in Figure 1c. The igniter is a NiCr bridge foil covered with an Al/CuO energetic film which produces a flame to ignite the ignition powder when stimulated by an electric current [17,18,19,20]. Boron/potassium nitrate (B/KNO_3_, BPN) was selected as the ignition powder and assembled in the powder chamber to avoid crushing.

### 2.2. Structure of the S&A Device

**Figure 2:**
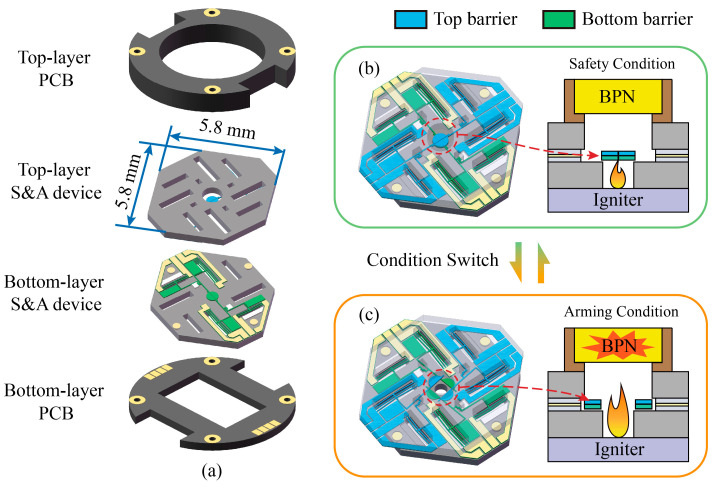
Structure of the S&A device. (**a**) Key components, (**b**) safety condition, and (**c**) arming condition.

The S&A device consists of two layers (top layer and bottom layer) of SOI wafers (50 μm device layer, 3 μm buried layer, and 400 μm handle layer) with an overall size of 5.8 × 5.8 × 0.9 mm^3^, and each layer has two semicircular barriers driven by bistable mechanism, as shown in Figure 2a. Two layers of the printed circuit board (PCB) and the S&A device form a sandwich structure to protect the chip’s structure. In addition, the electrodes of the two-layer chip are connected to the two PCB layers by gold wire leads and then connected by four pads around the PCB. When the S&A device is in a safe condition, the barrier covers the igniter. If the igniter is accidentally stimulated, the flame energy is blocked and cannot reach the BPN, as shown in Figure 2b. Otherwise, driven by an arming signal, the S&A device will switch to the armed condition to open the igniter. Flame energy is allowed to pass through the S&A device and ignite the BPN, as shown in Figure 2c.

### 2.3. Driving Principle of the S&A Device

**Figure 3:**
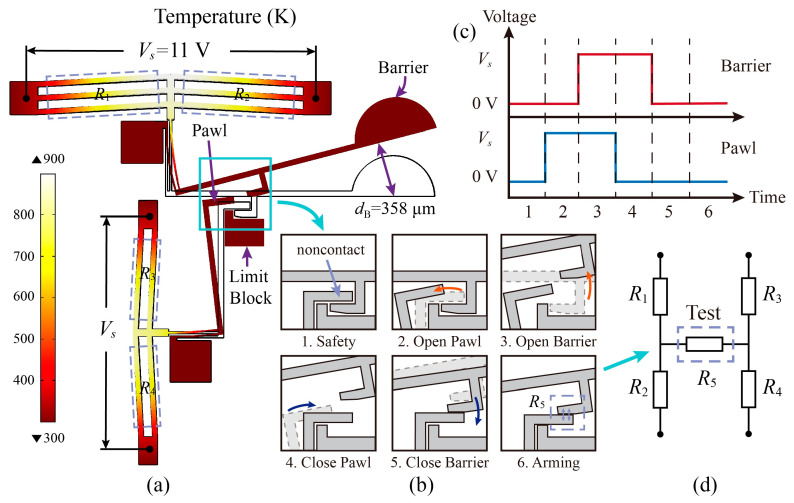
Driving Principle of the S&A device. (**a**) Simulation results, (**b**) operation process, (**c**) driven signal, and (**d**) circuit of feedback function.

A V-shape electrothermal actuator is selected as the drive unit for the S&A device; its drive theory has been extensively studied and, thus, is not described in this article. The displacement of the electrothermal actuator is too small to drive the barrier and a soft lever mechanism is used to enlarge the deformation. The S&A device uses four groups of bistable mechanisms to independently and synchronously control the double-layer barrier. Each bistable mechanism consists of two actuators driving a barrier and a pawl. The geometric parameters of the two actuators and the soft lever are shown in Table 1, and all the parameters are expressed in previous research [14]. Finite element simulations of the bistable mechanism are performed, and the results are shown in Figure 3a. When the applied voltage is set to 11 V, the barrier and the pawls can generate a displacement of 358 μm and 117 μm, which is sufficient for the mechanism.

According to a specific operation sequence, the bistable mechanism can control the engagement of the actuator and the pawl as shown in Figure 3b: (1) In the safety condition, the pawl as a safety lock engages with the barrier so that the barrier can open only a minute angle, and they are non-contact when no signal enters; (2) The pawl is opened to release the barrier’s movement; (3) The barrier is opened to the arming position; (4) The pawl is closed to prevent the barrier from returning to the safety position; (5) The barrier is closed to engage with the pawl; and (6) In the armed condition, the barrier is engaged with the pawl in the arming position, and the pawl is pressed against the limit block. The driven signal is two pulse signals with a phase difference, as shown in Figure 3c. Following a reverse operation process, the S&A device can recover to the safe condition.

The condition feedback function of the S&A device is realized on the basis of the bistable mechanism. The electrothermal actuators can be considered as a series connection of two resistors (*R*_1_ and *R*_2_ for the barrier actuator; *R*_3_ and *R*_4_ for the pawl actuator). In the armed condition, the barrier and the pawl are pressed tightly, which creates a contact resistance of *R*_5_. The circuit of the S&A device can be simplified as shown in Figure 3d. However, in the safety condition, the barrier and the pawl are separated and insulated from each other. Therefore, the *R*_5_ is infinitely large as an open circuit in the safety condition. Accordingly, the condition of the S&A device can be determined by testing the value of the *R*_5_.

## 3. Fabrication

**Figure 4:**
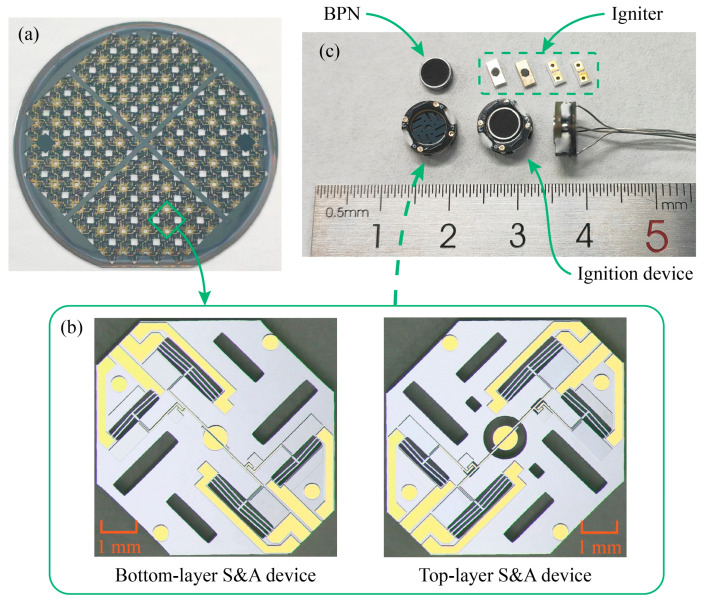
Fabrication of the ignition device. (**a**) Four-inch SOI wafer, (**b**) the S&A device chip, and (**c**) the ignition device.

The ignition device is prepared by an assembly of each component, including the S&A device, the igniter, and the BPN, where the S&A device is fabricated from a 4-inch SOI wafer by the etching process [14,15,16], as shown in Figure 4a, and each individual chip, as shown in Figure 4b. Two layers of the S&A device and two layers of the PCB are bonded layer-by-layer with epoxy resin, as shown in Figure 4c. The BPN is press-fitted into the powder chamber at a density of 1.47 ± 0.21 g/cm^3^ and placed above the S&A device. The igniter is fabricated on a ceramic substrate by a magnetron sputtering process, and the Al/CuO energetic film is filled by ink droplets [15,21]. All the independent parts are assembled layer by layer to form an ignition device.

## 4. Tests and Discussion

### 4.1. Test of the Bistable Function

**Figure 5:**
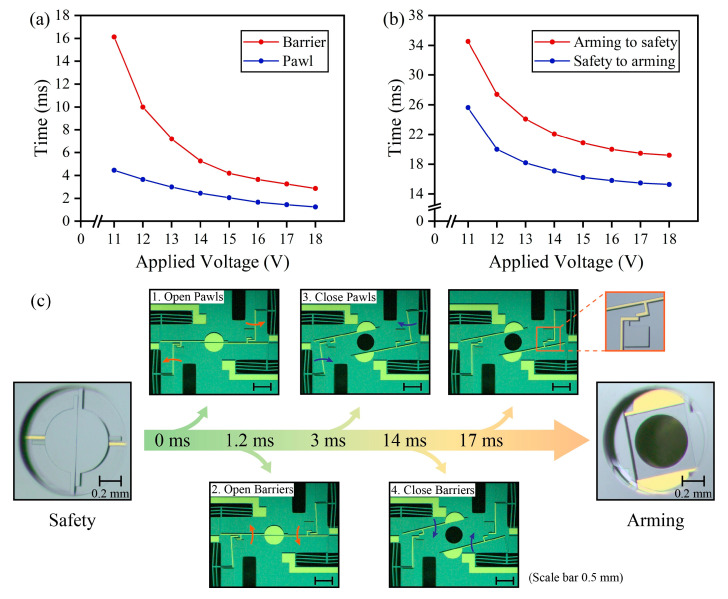
Test results of the bistable function. (**a**) Arming time versus applied voltage, (**b**) switching time versus applied voltage, and (**c**) the operation sequence of safe to armed with an applied voltage of 14 V.

According to the driving principle, the displacement and response time of the electrothermal actuator is directly determined by the applied voltage. The response process of the barrier and the pawl are recorded by a high-speed camera (5000 fps) from 11 V to 18 V, as shown in Figure 5. The arming time is the time it takes for the pawls and barriers to reach the arming position (pawl: 100 μm; barrier: 300 μm); the arming time as a function of the applied voltage is shown in Figure 5a. The switch time is the time required for the S&A device to switch to the arming or safety condition; the switch time as a function of the applied voltage is shown in Figure 5b. As the applied voltage increases (11 V to 18 V), the actuator takes less time to reach the armed position (barrier: 16.2 ms to 2.8 ms; pawl: 4.4 ms to 1.2 ms), and the switching time also decreases (armed to safe: 34.6 ms to 19.2 ms; safe to armed: 25.6 ms to 15.2 ms). The S&A device switches the condition through a specific operation sequence; the detailed process from safe to armed with an applied voltage of 14 V is shown in Figure 5c. The electrothermal actuator can increase the drive voltage to increase the heating power, but the cooling process of the actuator is natural cooling independent of the drive voltage. Therefore, in step 3, the barrier spends 11 ms waiting for the pawl to return to its initial position, taking the longest time of all the steps, so the switching time hardly decreases further with the applied voltage.

### 4.2. Test of the Feedback Function

**Figure 6:**
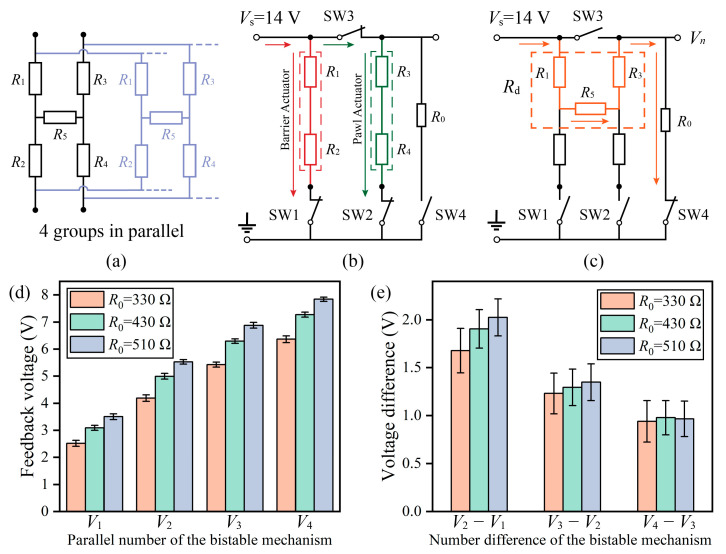
Test results of the feedback function. (**a**) Equivalent circuit of the S&A device, (**b**) the driving principle, (**c**) the feedback principle, (**d**) the test results for an *R*_0_ of 330 Ω, 430 Ω, and 510 Ω in different parallel numbers of the bistable mechanism, and (**e**) the voltage difference of the *V_n_*.

The S&A device consists of four identical bistable mechanisms, each containing a barrier actuator (*R*_1_ + *R*_2_) and a pawl actuator (*R*_3_ + *R*_4_), with the two actuators generating a contact resistance *R*_5_ in the armed condition. To simplify the control circuit, the four groups of bistable mechanisms are connected in parallel to drive the double-layer barrier synchronously, as shown in Figure 6a. To realize the drive and feedback function, four switches and a feedback resistor *R*_0_ are used to design the control circuit. The source voltage (*V_s_*) is set as 14 V. The driving principle of the circuit is shown in Figure 6b; switch 1 controls the barrier actuator, while switches 2 and 3 control the pawl actuator. Controlling the three switches to turn on/off in a specific sequence (Figure 3c) can switch the S&A device’s condition.

Only when all four bistable mechanisms are operating successfully will the S&A device switch to the safe or armed condition. Failure of any one set of mechanisms will cause the drive to fail. Therefore, the feedback function only needs to give feedback on the number of contact resistances *R*_5_ and does not need to give feedback on which specific *R*_5_ is having problems. The feedback principle is designed as shown in Figure 6c. Switches 1, 2, and 3 are set to the off state, and switch 4 is set to the on state. The current flows through the resistors *R*_1_, *R*_5_, *R*_3_, and *R*_0_ in turn. Since the resistance of *R*_5_ is much larger than *R*_1_ and *R*_3_, the current is small, and the actuator will not operate. The total resistance inside the S&A device is *R*_d_ = (*R*_1_ + *R*_3_ + *R*_5_)/*n* (*n* is the parallel number of the bistable mechanism). The *R*_d_ is variable with *n*, while the value of *R*_0_ is fixed. Therefore, the voltage division of *R*_0_ as the feedback voltage (*V_n_*) can give feedback on the resistance change of *R*_d_. The larger the voltage difference (*V_n_* − *V_n_*_−1_), the better the detection. *V_n_* − *V_n_*_−1_ can be expressed as:

(1) $$\left\{\begin{array}{c} k=\frac{R_{1}+R_{3}+R_{5}}{R_{0}}\\ V_{n}-V_{n-1}=V_{s}\frac{k}{k^{2}+\left(2n-1\right)k+n\left(n-1\right)}\left(n\geq 2\right)\\ {\left(V_{n}-V_{n-1}\right)}_{max}=V_{s}\frac{1}{2\sqrt{n^{2}-n}+2n-1}\left(k=\sqrt{n^{2}-n}\right) \end{array}\right.$$

when *n* is 4, *V_n_* − *V_n_*_−1_ reaches the minimum value. To make *V*_4_ − *V*_3_ reach the maximum value of 0.0718*V_s_* (1.01 V), *k* should be taken as 2$\sqrt{3}$. The measured value of *R*_1_ + *R*_3_ + *R*_5_ is about 1580 Ω, so *R*_0_ is taken as 330 Ω, 430 Ω, and 510 Ω for testing. The test results are shown in Figure 6d, where the *V_n_* can be seen to increase with the *R*_0_ and *n*. The voltage difference (*V_n_* − *V_n_*_−1_) is shown in Figure 6e. The *V*_4_ − *V*_3_ reaches the maximum value (0.98 V) when the *R*_0_ is taken as 430 Ω. Each drive of the S&A device causes a change in *R*_5_, which in turn changes *V_n_*. However, the change in *V_n_* is less than 0.15 V, which does not affect the detection. In the safety condition, the contact resistance *R*_5_ is not present and the value of the *V*_0_ is 0 V. The number of successfully operating bistable mechanisms can be detected by the feedback voltage.

### 4.3. Test of the Safety and Arming Functions

The safety and arming functions of the S&A device are used to prevent the BPN from being ignited by the igniter in the safe condition and to allow the BPN to be ignited in the armed condition. To test the safety capability of the device, the igniter was filled with different thicknesses of Al/CuO films to control the magnitude of the flame energy. The resistance of the igniter is 1 Ω, which can be excited by a 100 μF capacitor discharging at a charge voltage of 25 V. The safety and arming function of the double-layer barrier have been verified in a previous study [15], showing that it can block the flame energy of an Al/CuO film of 50 μm thickness and maintain the complete structure. However, if the flame energy is further increased, the barrier will deform in-plane and lose its safety function.

**Figure 7:**
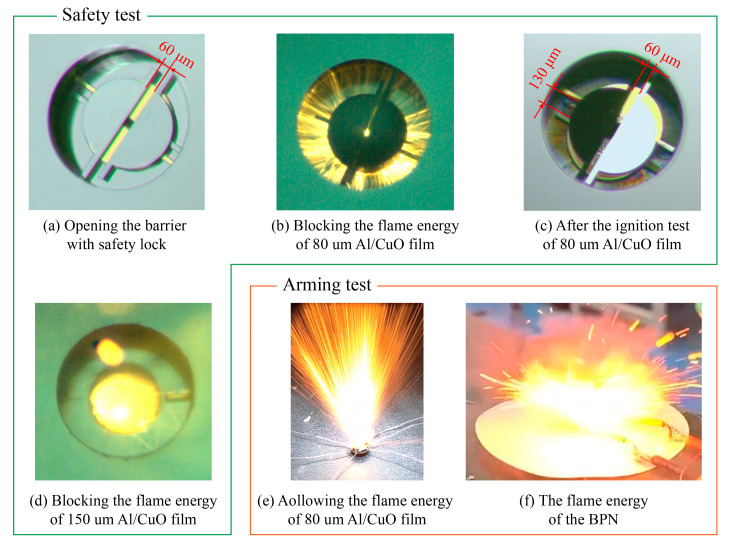
Test results of the safety and arming function.

Therefore, in this article, the pawl is designed as a safety lock to engage with the barrier at the safety position so that the barrier can only open a square gap of 60 μm × 60 μm with the safety lock closed, as shown in Figure 7a. To verify the improvement of the safety lock, the safety and arming functions were tested through Al/CuO film with an 80 μm, 100 μm, and 150 μm thickness; the test results are shown in Table 2. Four groups of tests were carried out for each thickness in the safety test and two groups in the arming test. When the thickness of the Al/CuO film was set to 80 μm and 100 μm, the flame energy was blocked by the barrier as shown in Figure 7b, and a rectangular gap of 60 μm × 130 μm was formed after the safety test, as shown in Figure 7c. Although the gap of the barrier was enlarged by the flame energy, the BPN was not ignited in the safety tests of the 80 μm and 100 μm thick Al/CuO films, so the safety lock significantly improved the safety function of the barrier. The safety lock can only restrain the in-plane deformation of the barrier, not the out-of-plane deformation. When the thickness of the Al/CuO film was set to 150 μm, the barrier broke out of the plane as shown in Figure 7d, causing the BPN to ignite. In the arming test, all of the flame generated by the igniter could flow through the S&A device and ignite the BPN, as shown in Figure 7e,f. The S&A device with a safety lock can realize the safety and arming function when the thickness of the Al/CuO film is set to 80 μm and 100 μm.

## 5. Conclusions

In this article, an intelligent MEMS safety and arming device with a condition feedback function was designed. The S&A device has a smaller size (5.8 × 5.8 × 0.9 mm^3^), lower driving voltage (11 V), and higher safety (safety lock) through an optimized structural layout. When the applied voltage was set to 14 V, the S&A device could switch the condition to armed within 17 ms or to safe within 22 ms. In addition, the device can give feedback on the device’s condition by the voltage division of an external resistor of 430 Ω. The feedback voltage can detect the number of successfully operating bistable mechanisms with a minimum voltage difference of 0.98 V. In summary, the safety lock can improve the safety function so that the device can realize the safety and arming function when the thickness of Al/CuO film is set to 80 μm and 100 μm. This MEMS S&A device with miniaturized structure and intelligent actuation is suitable for applications in micro combat platforms such as unmanned aerial vehicles (UAV).

## Figures and Tables

**Table 1:** Geometrical parameters of the electro-thermal actuator and the soft lever mechanism.

Item	Barrier Actuator	Pawl Actuator	Unit
Width (*w*)	35	35	μm
Length (*L*)	1900	1500	μm
Thickness (*h*)	50	50	μm
Angle (*θ*)	3	3	°
Number of beams	3	2	None
Width of the soft lever (*w*′)	14	14	μm
Length of the soft lever (*L*′)	500	350	μm
Distance of the soft beams (*L*_d_)	30	30	μm
Enlarged proportion	55	33	None

**Table 2:** The test results of the safety and arming function.

Thickness of Al/CuO Film	Charge Quantity of Al/CuO Film	Ignition Test in Safety Condition	Charge Quantity of Al/CuO Film	Ignition Test in Arming Condition
80 μm	0.47 mg	Safety	0.42 mg	Ignition
	0.42 mg	Safety	0.41 mg	Ignition
	0.43 mg	Safety	/	/
	0.42 mg	Safety	/	/
100 μm	0.52 mg	Safety	0.50 mg	Ignition
	0.51 mg	Safety	0.53 mg	Ignition
	0.51 mg	Safety	/	/
	0.54 mg	Safety	/	/
150 μm	0.74 mg	Ignition	0.74 mg	Ignition
	0.76 mg	Ignition	0.76 mg	Ignition
	0.73 mg	Ignition	/	/
	0.74 mg	Ignition	/	/
